# Johannes [Jan] Vermeer (1632–1675). The Astronomer (1668)

**DOI:** 10.3201/eid1001.AC1001

**Published:** 2004-01

**Authors:** Polyxeni Potter

**Figure:**
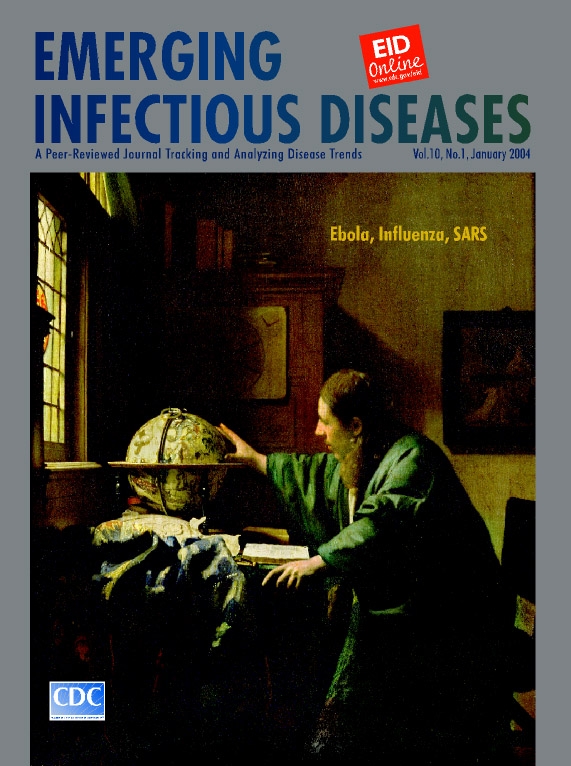
**Johannes [Jan] Vermeer (1632–1675). The Astronomer (1668).** Oil on canvas (51 cm x 45 cm). Musée du Louvre, Paris, France. Photo: Erich Lessing/Art Resource, New York

 “What is it about Johannes Vermeer?” contemporary art lovers and historians ask. The enigmatic painter, apparently well known in his day, lapsed into obscurity after his death only to surface again in the 19th century and capture the imagination and esthetic taste of modern times. Even though he produced no more than 40 paintings, their originality and refinement place him among the greatest 17th-century Dutch artists (*1*).

Vermeer’s life story can only be patched together from public records, and no portrait is available of his physical appearance. He was sufficiently trained in his trade to belong to an artists’ guild and was esteemed enough by his colleagues to be twice appointed guild leader. Nonetheless, financial hardship, aggravated by his inability to support a brood of 15 children, 11 of whom survived to adulthood, limited his artistic output. He made little money from his paintings and died poor at age 43 (*2*). “Because of…the large sums of money we had to spend on the children, sums he was no longer able to pay,” his wife lamented after his death, “he fell into such a depression and lethargy that he lost his health in the space of one and a half days and died” (*3*).

Though untraveled, Vermeer was well connected with other artists, including Gerald Ter Borch and Dirk van Baburen, and might have been influenced by Caravaggio and Carel Fabritius, the brilliant student of Rembrandt. Vermeer’s work is also reminiscent of 15th-century Flemish art, especially in the use of color and meticulous detail; however, he brought to these elements unique sensitivity and novelty (*4*).

During the 17th century, the arts broke away from classical style into genre (scenes of everyday life). Vermeer’s work assumed the domestic intimacy of the Dutch school yet moved beyond its monochromatic, evenly lit, raucous gatherings. Although more than any of his contemporaries Vermeer saw poetry in everyday activities (The Lacemaker, The Procuress, The Milkmaid), his characters had an air of introspection and seemed to be engaged in more than the activities themselves. In scenes of extraordinary simplicity and clarity, he placed solitary figures, whose dimensions were sometimes enlarged in relation to the surroundings, within the confines of carefully constructed spaces (*1*). Then, elaborating on textures and details, he used light to peer through the external trappings into the soul.

While the arts were abandoning classical themes for scenes of everyday life, the sciences were inventing new methods of inquiry, dispelling the shadows of antiquity to view the world through a lens. Newton was making the first reflecting telescopes, Louis XIV was building an observatory in Paris, and Huygens had detected the first moon of Saturn (*5*,*6*). An innovator himself, Vermeer was likely acquainted with the science of his time and is often linked with Anton van Leeuwenhoek, Dutch inventor of the microscope. Van Leeuwenhoek, a merchant who sold cloth not far from Vermeer’s abode in Delft and who later became the court-appointed executor of the artist’s will, discovered through his microscope the cellular nature of spermatozoa and bacteria and was skilled in navigation, astronomy, and mathematics. Van Leeuwenhoek, who was born the same year as Vermeer, may have inspired both The Astronomer (on this month’s cover of Emerging Infectious Diseases) and its companion, The Geographer (c.1668–1669) (*3*).

The Astronomer harmonizes space, color, and light to convey a single human activity, a unified moment in time. Perfectly staged, the scene is a subtle composite of interlocking diagonal, rectangular, or elliptical fields and has no empty or undefined surface. The composition is not narrative but rather forms the context of a sole figure, frozen in a pose of profound preoccupation.

Like many Vermeer characters, the astronomer is placed near a window on the onlooker’s left, which casts a glow on the man of science, revealing youthful freshness, sudden insight, and nervous anticipation. Expressive hands define the geometric space between the sympathetic figure and the celestial globe (drafted by Dutch cartographer Jodocus Hondius in 1600) and drive the forward movement of the body. The desk, framed by a thick tapestry, holds an astrolabe (precursor of the sextant) and a book. On the wall is a circular figure with radial lines.

The moment of discovery reflected on the astronomer’s face captures centuries of human fascination with the universe. The generic physiognomy, unremarkable features, untended hair, and drab attire draw the eyes to the illuminated face of the thinker, in a room where the only light is that of knowledge.

A model to all stargazers, old and new, Vermeer’s scientist reaches beyond the globe at hand into the mysterious continuum of time and space, charting, measuring, counting, categorizing, naming, recording. His contemporary counterpart, whether an astronomer exploring the cosmos or a biologist investigating the microcosm, is still guided by the light of discovery. Uncharted in Vermeer’s days, the spatial distribution of disease follows the evolution over time of agent, host, and environment and is the domain of those today who trace the time-space continuum of emerging pathogens, from Ebola to influenza and SARS.
